# Prognostic role of immunohistochemical PTEN (phosphatase and tensin homolog) expression and PTEN (rs701848) genotypes among Egyptian patients with different stages of colorectal cancer

**DOI:** 10.7150/jca.97553

**Published:** 2024-07-29

**Authors:** Heba Ahmed Osman, Mohammed H. Hassan, Abdelaziz Mostafa Toema, Amira A. Abdelnaby, Mahmoud A. Abozeid, Mohamed Abdelshafy Mohamed, Abdelkader Ahmed Hashim, AbdAlraheem Husein, Abdelazeem E. Ahmed, Sameh Salaheldin Elsayed, Sherief El-Ghannam, Marwa Abdelhady, Ghada M. Abdelrazek

**Affiliations:** 1 Department Of Tropical Medicine and Gastroenterology, Qena Faculty of Medicine, South Valley University, Qena, Egypt.; 2 Department Of Medical Biochemistry, Faculty of Medicine, South Valley University, Qena, Egypt.; 3 Department of Biochemistry, Clinical Pharmacy Program, South Valley National University, Qena, Egypt.; 4 Department of Oncology and Nuclear Medicine, Faculty of Medicine, Al-Azhar University, Assiut, Egypt.; 5 Oncology Center, John's Hopkins Aramco Healthcare, KSA.; 6 Department of Pathology, Faculty of medicine, Sohag University, Sohag, Egypt.; 7 Department of Restorative Dentistry and Basic Medical Sciences, Faculty of Dentistry, University of Petra, Amman 11196, Jordan.; 8 Department of General surgery, Faculty of medicine, South Valley University, Qena, Egypt.; 9 Department of General surgery, Faculty of Medicine, South Valley University, Qena, Egypt.; 10 Department of Internal Medicine, Faculty of Medicine, South Valley University, Qena, Egypt.; 11 Department of Radiodiagnosis, Faculty of Medicine, South Valley University, Qena, Egypt.; 12 Department of Clinical Pathology, Faculty of Medicine, Al-Azhar University, Assiut 71524, Egypt.; 13 Department of medical biochemistry faculty of medicine, Al-AZHAR university Assiut, Egypt.; 14 Department of Clinical Pathology, Damietta Faculty of Medicine, Al-Azhar University, New Damietta City, Egypt.; 15 Department of Internal Medicine, Faculty of Medicine, Luxor University, Luxor, Egypt.; 16 Department of Radiodiagnosis, Faculty of Medicine, South Valley University, Qena, Egypt.

**Keywords:** CRC, PTEN, immunohistochemical, genotype, AJCC

## Abstract

Colorectal cancer (CRC) is the third most frequent cancer worldwide and the second major cause of cancer-related death. Thus, we attempted to ascertain the relationship between the genotype and allele frequencies of phosphatase and tensin homolog (PTEN) and immunohistochemical PTEN expression with clinicopathological characteristics in patients with CRC. 150 individuals were allocated into two groups for this cross-sectional randomized case-control study: Group I consisted of 100 patients with histopathologically proven CRC of various stages. Group II: Fifty healthy volunteers. Genetic analysis of PTEN (rs701848 T / C) single nucleotide polymorphism (SNP) was performed using TaqMan^TM^ assays and real-time PCR, while PTEN expressions were assessed using immunohistochemical staining. PTN SNP genotypes and alleles did not significantly differ between CRC patients and controls. PTEN expression was lost in 28% of CRC patients, while all healthy controls exhibited PTEN expression. Negative PTEN expression was present in 16 (80%) of stage IV CRC cases, 9 (23.7%) of stage III cases, 3 (37.5%) of stage II cases, and none of stage I cases. It was shown that PTEN expression was weakly positive, moderately positive, and strongly positive in 15, 10, and 9 (respectively) cases of CRC stage I. However, the expression was only weekly positive in 4 (20%) of the patients in stage IV. In the stage IV group, neither moderately nor strongly positive PTEN expressions were found. So, Among Egyptians, the emergence or course of colorectal cancer is unrelated to the PTEN gene mutation. However, the formation and progression of CRC may be influenced by weak or lost PTEN expression.

## Introduction

There is a growing prevalence of colorectal cancer (CRC) in Egypt's younger population. CRC ranked sixth in Egypt with approximately 4% of all cancer cases, behind pulmonary, breast, prostate, hepatic, and bladder cancer 1. Understanding the molecular evolution of CRC as well as the genetic and environmental risk factors can help prevent and treat the disease 2. PTEN (phosphatase and tensin homolog deleted on chromosome ten) is a tumor suppressor gene found at 10q23 that was initially identified in the late 1990s 3. PTEN's primary function is to dephosphorylate phosphatidylinositol triphosphate to phosphatidylinositol bisphosphate, which blocks PI3K function. Few studies have examined PTEN's role as a predictor or prognostic factor in colorectal cancer (CRC); as a result, the relationship between PTEN and CRC remains unclear. PTEN loss of function can alter the PI3K pathway in colorectal cancer and may play a role in the malignant evolution of benign lesions 4-5. There were only two previous studies conducted in Egypt to assess the role of PTEN in CRC patients, and they concluded that PTEN loss may be linked to a lower chance of a favorable result for CRC patients and may potentially be associated with the formation of CRC. 5-6. Therefore, we aim to determine the association between PTEN genotype and allele frequencies as well as immunohistochemical PTEN expression and clinicopathological features in patients with different stages of CRC to predict the patient's outcome based on the PTEN status.

## Materials and Methods

### Participants and study design

This cross-sectional randomized case-control study has been conducted on 150 participant from 1st January 2022oups: Group I: 100 patients manifested with bleeding per-rectum, chronic constipation, intestinal obstruction, weight loss, anaemia and/or chronic diarrhea with histopathologically proven colorectal cancer who attended the endoscopy unit of the Tropical Medicine and Gastroenterology Department, Internal Medicine and Surgical departments, Qena Faculty of Medicine, South Valley University, in the period from 1^st^ January 2022, to 30^th^ May 2023. Group II: 50 healthy volunteers.

All participants were subjected to a complete history-taking and full clinical examination. All patients, after histopathological confirmation of colonic carcinoma, were referred to the General Surgery Department for surgical resection.

Patients with a previous diagnosis of CRC who received chemotherapy or radiotherapy, cancer anywhere in the body except CRC, recurrent cancer colon, inflammatory bowel disease, and any disease in the colon except CRC were excluded from the study. CRC is staged according to the American Joint Committee on Cancer (AJCC) staging system 7.

### Investigatory workup

Colonoscopy evaluation: All participants who presented with bleeding per rectum, chronic diarrhea, chronic constipation, anemia (after exclusion of blood disease), weight loss, or intestinal obstruction underwent a lower endoscopic examination after proper bowel preparation using the PENTAX-EPK-i5000 endoscope. Under sedation with intravenous midazolam 5 mg by an endoscopist with more than five years' experience, an endoscopic punch biopsy was taken for histopathological and PTEN immunohistochemical assessment.

Baseline liver functions, kidney functions, CBC, prothrombin time, and tumor markers (CEA and CA19-9) assays.

CT chest, abdomen and pelvis was done to detect a local and distant metastasis.

Pelvic MRI was performed on a 1.5 T magnet (Philips Acheiva, Guildford Business Park, Guildford, Surrey, Netherlands) with pelvic phased array coil and rectal gel administration. All patients underwent imaging while in the prone position following the placement of a small Foley catheter in the rectum and insufflation of approximately 200 to 300cm of room air. A sagittal fast-spoiled gradient echo sequence was used to localize the lesion. This was followed by axial, conventional, and spin echo T2-weighted images. Coronal and sagittal fast spin echo T2- weighted images were obtained. Post-contrast T1, Diffusion-weighted images. All images were interpreted by the same radiologist. Reporting was made regarding the depth of invasion of the rectal wall, adjacent organ involvement, and the presence of lymphadenopathy.

#### MRI interpretation criteria: A-T staging interpretations

• T1 was staged if the tumor was confined to the mucosal layer of the rectal wall.

• T2 was staged if there was an invasion of the rectal layer up to the muscularis propria with no penetration of the muscularis propria or perirectal fat.

• T3 was staged if there was an invasion of all rectal layers with perirectal fat infiltration yet without pelvic organ involvement.

• T4 was staged if there was an invasion of the mesorectal fascia and visceral peritoneum or surrounding organ infiltration.

#### B- Lymph node staging interpretations

**Figure 1:**
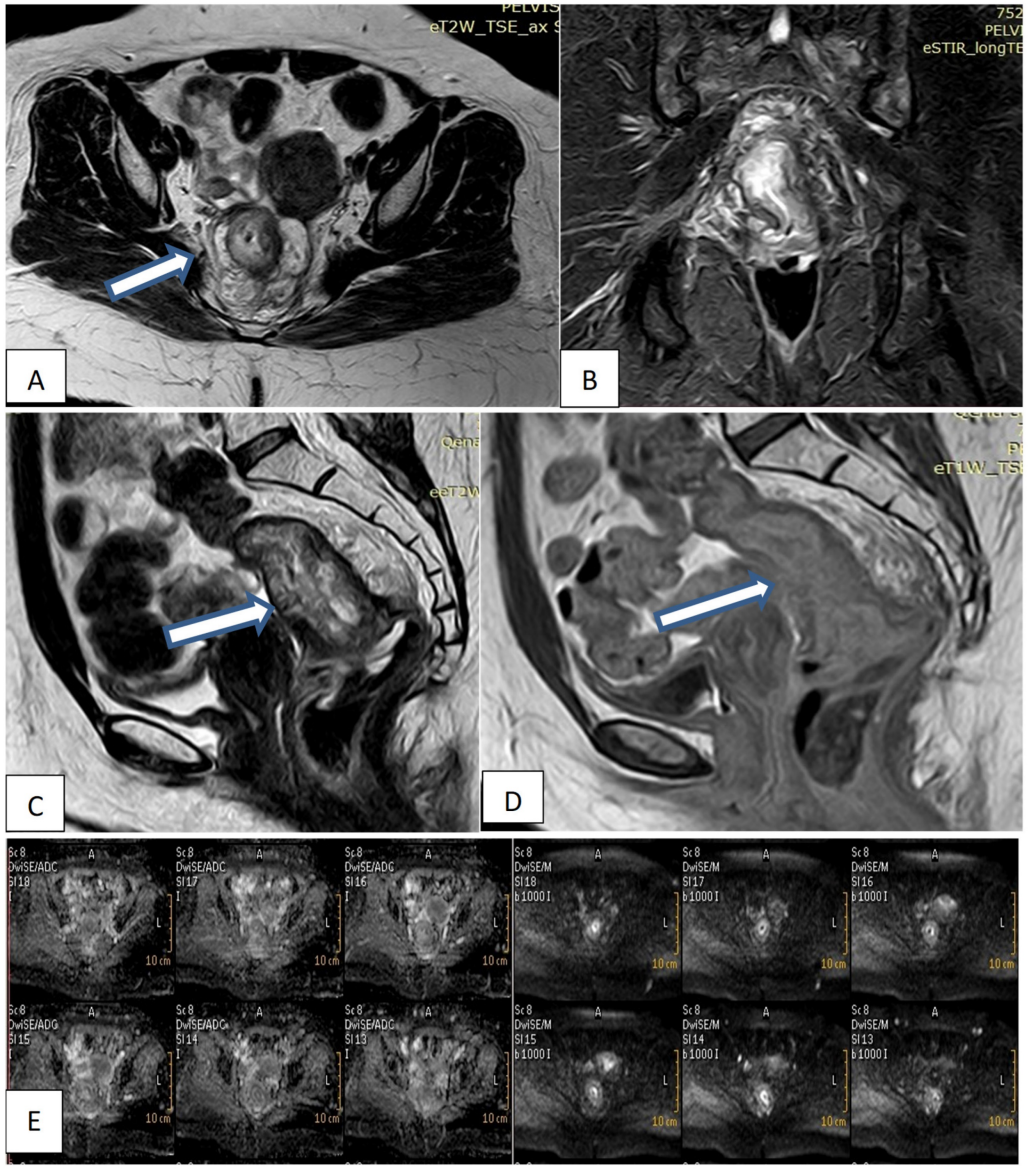
Female patient 47years old with mucinous rectal mass. (A, B, C) Axial & sagittal T2W & coronal STIR MRI images show transmural circumferential thickening about 7.5cm length & 6cm from the anal verge, invading muscularis propria & reaching perirectal fat, not infiltrating levator ani (T3c). (D) Sagittal T1W1 with contrast shows faint enhancement. (E) DWI & ADC images show restricted tumor tissue.

• N0 was diagnosed if there was no lymph node metastasis.

• N1 was diagnosed if there was metastasis in one to three lymph nodes.

• N2 was diagnosed if there was metastasis in four or more perirectal lymph nodes **(Fig. 1)**.

### Genotyping of PTEN (rs701848 T / C) by TaqManTM assays and real-time PCR

PTEN (rs701848 T / C) genotyping using real-time PCR and TaqManTM assays: Each participant had five cc of venous blood drawn, which was then evacuated into tubes containing EDTA and kept at -80 °C until the time of the genetic analysis. DNA was extracted from whole blood by following the manufacturer's instructions (QIAamp DNA blood mini kits for genomic DNA purification, catalogue no. 51104, Hilden, Germany). Using the following primers (forward 5'CATAGTGCTCCCCCGAGTTG3' & reverse 5'CCGCTTAAAATCGTATGCAGTCT3') and TaqMan^TM^ assay fluorescent-labeled probes (VIC/FAM) TGCTCCCCCGAGTTGGGACTAGGGC[T/C] TCAATTTCACTTCTTAAAAAAAATC, provided by Thermo Fisher Scientific, Waltham, MA, USA, catalog number 4351379, specifically designed to discriminate the variant of the PTEN T/C (rs701848) polymorphism. A real-time PCR thermocycler (Rotor-Gene Q, Applied Biosystems, Foster City, CA, U.S.A.) was utilized for the automated genotyping of DNA samples in compliance with the manufacturer's methodology on purified genomic DNA.

**Figure 2:**
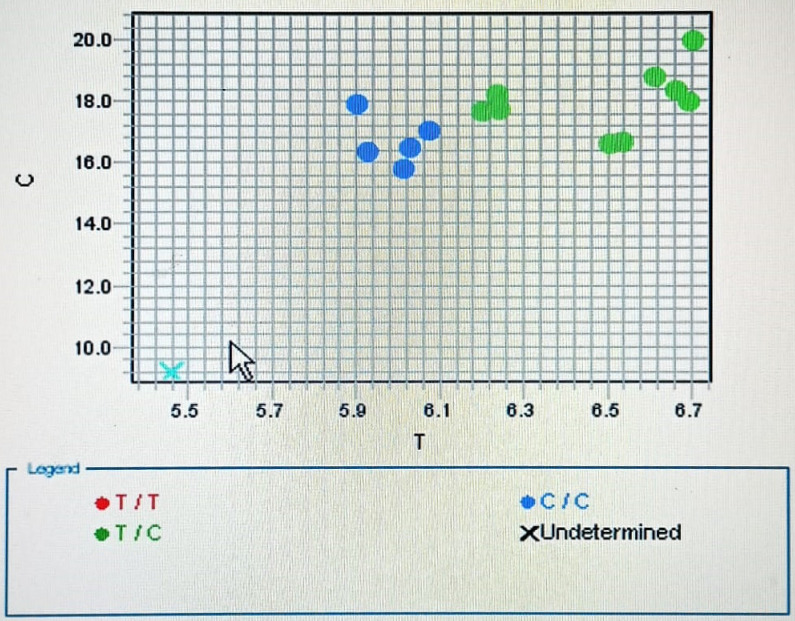
Allelic discrimination plot of PTEN T/C (rs701848) polymorphism using real-time PCR.

In short, the following were the thermocycler's conditions: As demonstrated in previously published work 8, 50°C for 2 minutes, 95°C for 10 minutes, and 50 cycles of 92°C for 15 seconds and 60°C for 90 seconds were conducted. The allele discrimination plots for rs701848 T/C were displayed in **(Figure 2)**.

### Histopathological and immunohistochemical examination

The study included tissue samples of 100 cases of CRC confirmed by endoscopic biopsy, then removed surgically, and 50 cases of healthy controls. Tissue samples obtained by endoscopic biopsy.

100 Paraffin-embedded tissue blocks of CRC specimens and 50 blocks from healthy controls were prepared from specimens removed surgically and by endoscopy, which were previously fixed with formalin for 24 hours. Sections 4 um thick were obtained, and for the diagnosis and grading of CRC cases, they were stained with hematoxylin and eosin stains. Staging was done according to AJCC 8^th^ edition.

The sections were deparaffinized in xylene for 20 minutes and then placed in descending grades of alcohol (100%, 80%, and 70%) for 2 minutes for each for rehydration. After that sections were rinsed in distilled water. For 10 minutes, 3% hydrogen peroxide was used to block endogenous peroxidase activity. Antigen retrieval was accomplished by boiling sections in citrate buffer solution PH 6 for 20 minutes.

Tissue sections were incubated with mouse anti-PTEN monoclonal primary antibody (Catalog No. MC0531, clone PTEN/2110, Medaysis, USA) at a 1/200 dilution overnight at 4 degrees Celsius. After that, we added goat anti-Mouse secondary antibody to tissue sections at room temperature for 45 minutes, then rinsed in distilled water. Diaminobenzidine (DAB) chromogen was utilized on tissue sections until a positive control exhibited brown staining. Sections were counterstained with Mayer's hematoxylin, dehydrated in ascending grades of alcohol, cleared with xylol, and eventually, DPX and cover slipped.

Sections from ductal carcinoma of the breast were used as a positive control. Every immunohistochemical staining run consists of a negative control using the same procedures as above but with phosphate buffer solution added in place of the primary antibody.

### Scoring and interpretation of immunoreactions

PTEN expression appeared as brownish cytoplasmic staining. Scoring system using the sum of the extension area and intensity was used to each of the IHC-stained regions. Staining intensity was determined as 0,1,2 and 3 points representing negative, mild, moderate, and strong brown staining, respectively. The immunoreactive areas were scored as 1: <25%, 2: 26-50%, 3:51-75% and 4: >75%. A total combined score was obtained. It was categorized as 0, +, ++ and +++. Final scores 2 and more were considered positive 9.

### Statistical analysis

Data were analyzed using Statistics Package for Social Sciences (SPSS) version 26. Normality test (Kolmogorov-Smirnov & Shapiro-Wilk test) was performed and data (age, Hgb and PLT) were normally distributed. In contrast, data for (WBCs, PT, PC, INR, Create, ALT, AST, albumin, and bilirubin) were not normally distributed. Continuous data were expressed as mean±standard deviation (Mean±SD) or median and Interquartile range (Median (IQR). Differences between the two groups were detected using independent samples T- test for parametric data and Mann-Whitney test for non-parametric data. Nominal data were expressed as percentage; differences between the two groups were detected using the Chi square test. The studied SNPs followed the Hardy-Weinberg (HW) equation. To achieve 80% power and a 5% level of confidence in the results (type I error), we adjusted the sample size.

## Results

This randomized case-control study has conducted on 100 patients with histologically confirmed colorectal cancer with different grades and stages, compared with fifty age and sex matched healthy controls.

### Demographic data of studied groups

**Figure 3:**
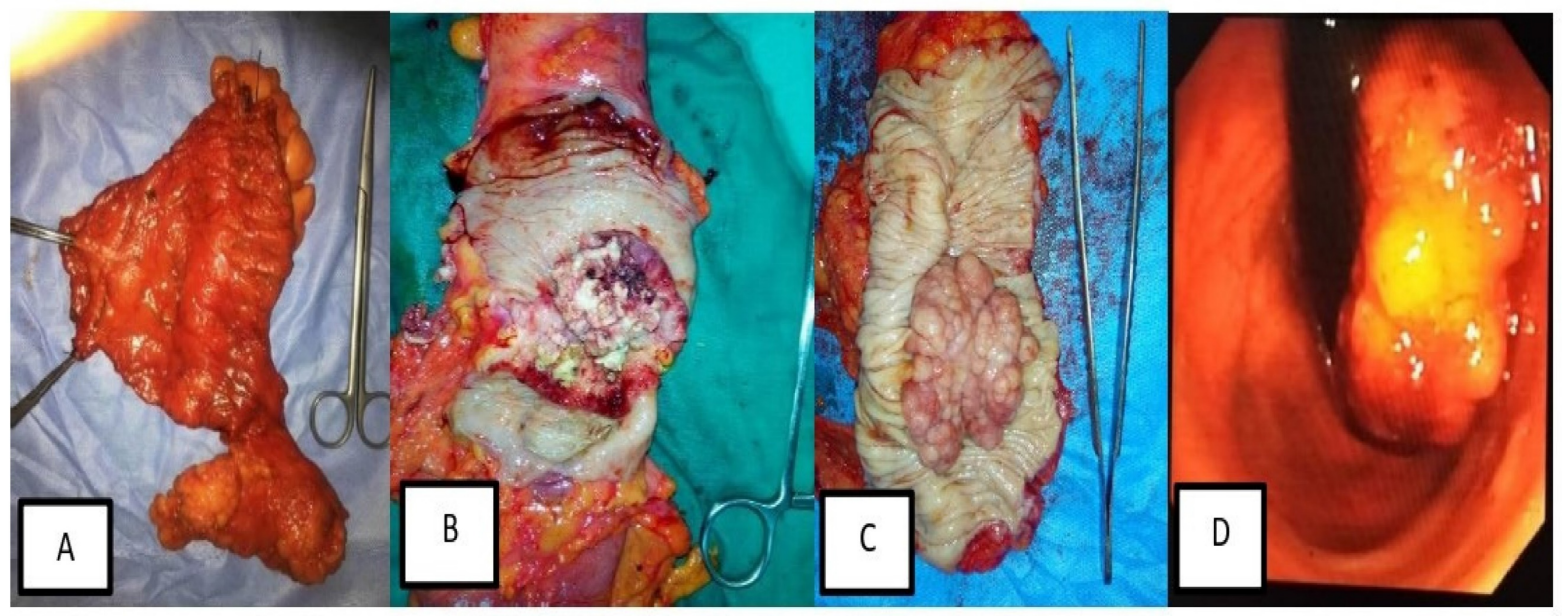
Different forms of surgical and Endoscopic lesions in patients with different stages of colorectal cancer. (A) Radical left hemicolectomy specimen with its mesentery and high vascular ligation of inferior mesenteric artery and vein. (B) Large ulcerating mass occupying more than 2/3 of the colon circumference. (C) Large Cauliflower-like mass in radical sigmoidoscopy. (D) Endoscopic view of Cauliflower, fungating lower rectal mass.

Of the total 100 patients with **CRC**,** 48** patients** (48%)** had rectosigmoid cancer,** 20 (20%)** had cancer caecum**, 17 (17%)** had ascending colon cancer and** 15 (15%)** had descending colon cancer. There was a male preponderance with the male patients represented** 60%** of patients. The mean age of patients at presentation was** 48.87±12.91** years** (Fig. 3)**.

The main presenting manifestations of patients with colorectal cancer were bleeding per-rectum, chronic constipation, and weight loss.

Positive family history of **CRC** was detected in **6%** of cases, while positive family history of cancer elsewhere in the body other than **CRC** was detected in **4%** of cases.

PTEN (rs701848) genotype **TT** was detected in **10 (10%)** of CRC cases and **6 (12%)** of controls, **TC** was detected in **60 (60%)** of cases and **30 (60%)** of healthy volunteers, and **CC** genotype was detected in **30 (30%)** of cases and **14 (28%)** of controls, with non-significant p value **(p=0.918) (Table 1)**.

### Laboratory data of studied participants

Regarding the complete blood count of patients with CRC, mean hemoglobin level was **10.4±1.62** with significant difference compared with healthy controls **11.14±1.49**, **(p=0.008)**.

Regarding liver function tests, patients with CRC showed marked reduction of mean albumin level and increased in mean bilirubin level **3.83±0.64** and **1.56±2.03**, respectively, compared with **4.22±0.57** and **0.91±0.34**, respectively in healthy controls. The median albumin level among CRC patients was **3.8 (3.5-4.1)**, while the median bilirubin level was **1.1 (2.96-1.7)**. Conversely, the healthy control group's median albumin and bilirubin levels were, respectively, **4.1 (3.9-4.7)** and **0.99 (0.67-1.1) (p=0.001,** each**)**.

The rest of the laboratory data is detailed in **Table 1**.

### Genotypes and allele frequencies of PTEN (rs701848) genotype among study groups

The comparison between the two study groups regarding **PTEN** genotypes and allele frequencies, showed lower frequencies of wild genotype **TT** with equal frequencies of heterozygous mutant genotype **TC** and higher frequency of homozygous mutant genotype **CC** in CRC patients versus controls with non-significant p. value** (10%, 60% and 30% versus 12%, 60% and 28% respectively, p < 0.918).**

Using the dominant model, genotypes **(TT+TC)** were less prevalent among the CRC cases versus controls with no significant difference **(70% vs 72%, p< 0.8, OR: 0.907, 95%, CI: 0.428-1.923)**.

On the other side, using the recessive model, genotypes **(TC+CC)** were more prevalent among CRC cases compared with control with no significant p value **(90% vs 88%, p< 0.708, OR: 0.815, 95%, CI: 0.278-2.386)**.

In addition, there was non-significantly higher allele T and lower allele C frequencies among the controls compared to CRC cases** (42% and 40% vs 58% and 60%, respectively; p<0.740).** Indicating that, T allele did not carry any risk of CRC development and C allele did not have any protective value among Egyptian patients with OR: 0.921, CI: 0.565-1.499** (Table 2).**

### PTEN genotypes and allele frequencies with the histologic grading and AJCC staging of CRC

Regarding genotypes frequencies and histologic grade of CRC, no significant relation was detected between different genotypes and either early or late histologic grade of cancer** (p=0.102)**. Also, no significant association was detected between T or C alleles frequencies and early or late histologic grade of cancer **(p=0.288)**.

Comparison between early and late stage of cancer, neither PTEN genotypes nor allele frequencies showed significant association with early or late stages of CRC **(p=0.092 and 0.365; respectively)**. Indicating that T allele has no role in differentiating between early or late histologic grade of cancer. At the same time T allele has no role in differencing between early or metastatic colorectal cancer **(Table 3)**.

### Immunohistochemical PTEN expression in cases of Colorectal cancer and control

All enrolled cases in the study were examined for PTEN immunohistochemical expression. PTEN expression was localized mainly to the cytoplasm of colonic cells both in CRC cases and controls.

As shown in **Table 5**, there was a highly significant difference between expression of PTEN in CRC cases and normal healthy controls, **28%** of patients with CRC lost PTEN expression versus none of healthy controls loss immunohistochemical PTEN expression.

Concerning positive PTEN expression in CRC cases versus controls, **32%** of patients showed **1+ PTEN** expression **(weakly positive)**, **20%** showed **2+ PTEN** expression **(moderately positive)**, and **20%** showed **3+ PTEN** expression **(strong positive)**; versus **8%** of controls showed **1+ PTEN** expression, **32%** showed **2+ PTEN** expression and **60%** showed **3+ PTEN** expression; indicating that immunohistochemical PTEN expression could have a protective role and loss of its expression carry high risk of cancer development with significant p value **(p=0.001) (Table 4)**.

### Association between PTEN expression and the clinicopathological parameters in CRC patients

Histologically, cases of CRC were classified as **33** cases of colorectal adenocarcinoma **(33%)** grade **I**, **44 (44%)** grade **II** and **32 (32%)** grade **III**, Among the studied cases, **70 (70%)** was conventional adenocarcinoma and the rest of the cases were mucinous ones.

According to AJCC (American Joint Committee Cancer) staging system 8^th^ edition **24(24%)** of CRC cases invades submucosa only **(T1)**, **44 (44%)** were **T2** and the rest was staged as **T3**. No cases were staged as **T4** among the collected samples. Between CRC cases, **45(45%)** were node negative (N0). Cases of CRC were classified into **34(34%)** stage **I**, **8** cases **(8%)** stage **II**, **38 (38%)** stage **III** and **20** cases **(20%)** were stage **IV**. No significant relation was detected between PTEN expression and age, gender, histologic type, and histologic grade of colorectal cancer.

No statistically significant relation was detected between PTEN expression and age, gender, site, histologic type and histologic grade of CRC cases.

There was a statistically significant association between PTEN expression and T stage of CRC cases (p=0.027*). Loss of PTEN expression was detected in **16 (50%)** of cases staged as stage **T3** and **4 (16.7%)** of cases staged as **T1**.

Another significant relationship was detected between PTEN expression and nodal metastasis (p=0.030*), PTEN expression was negative, weakly positive, moderately positive and strongly positive in **6 (13.3%)**, **15 (33.3%)**, **14 (31.1%)** and **10 (22.2%)** of cases with negative nodal metastasis **(N0)** respectively, the expression was also negative, weekly positive, moderately positive and strongly positive in **14 (46.7%)**, **8 (26.6%)**, **4 (13.3%)** and **4 (13.3%)** of cases staged as **N1** respectively. Cases of CRC staged as **N2** showed negative, weak positive, moderately positive, and strongly positive expression in **8 (32%)**, **9 (36%), 2 (8%)**,** and 6 (24%)** of cases, respectively.

Regarding AJCC stage groups, there was statistically significant relationship between staging and PTEN immunohistochemical expression. Negative PTEN expression was detected in **16 (80%)** of cases of CRC staged as **stage IV**, **9 (23.7%)** of **stage III** cases, **3 (37.5%)** of **stage II** cases and no loss of expression was detected in **stage I** cases.

PTEN expression was also weakly positive, moderately positive, and strongly positive in **15 (44.1%)**, **10 (29.4%)**, **9 (26%)** respectively of cases of CRC **stage I**.

**Figure 4:**
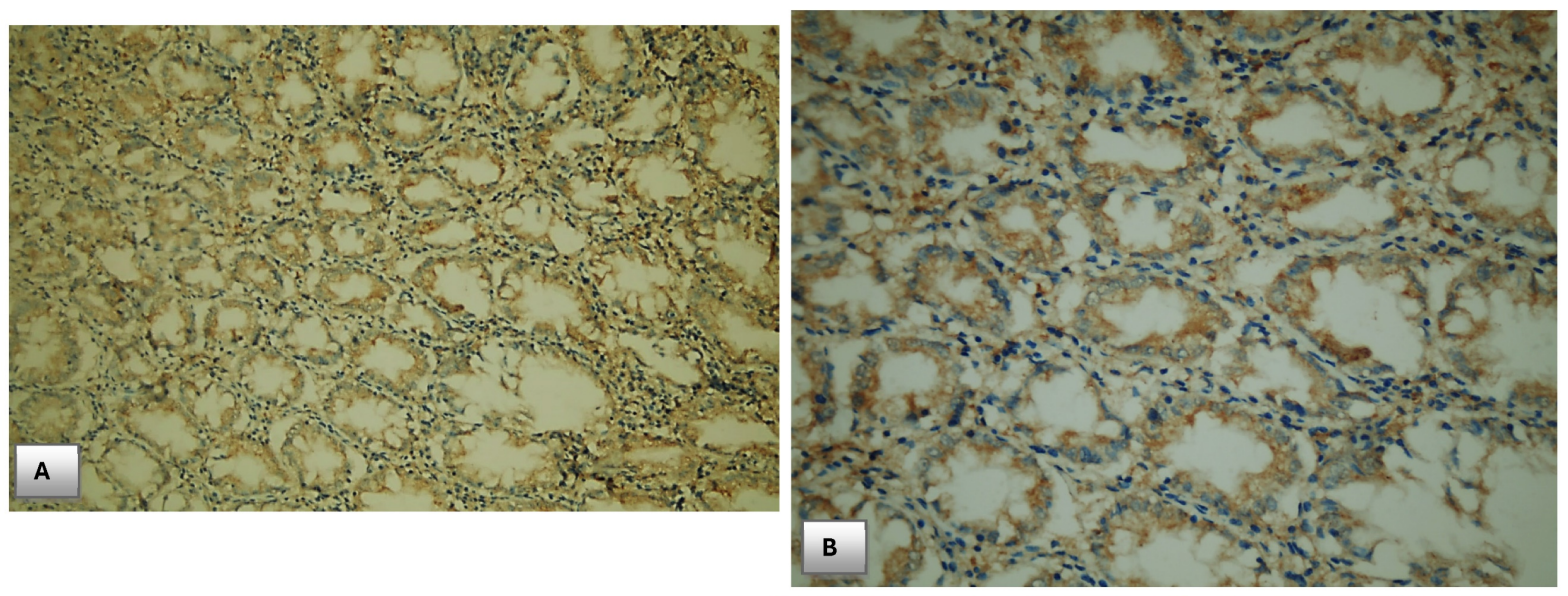

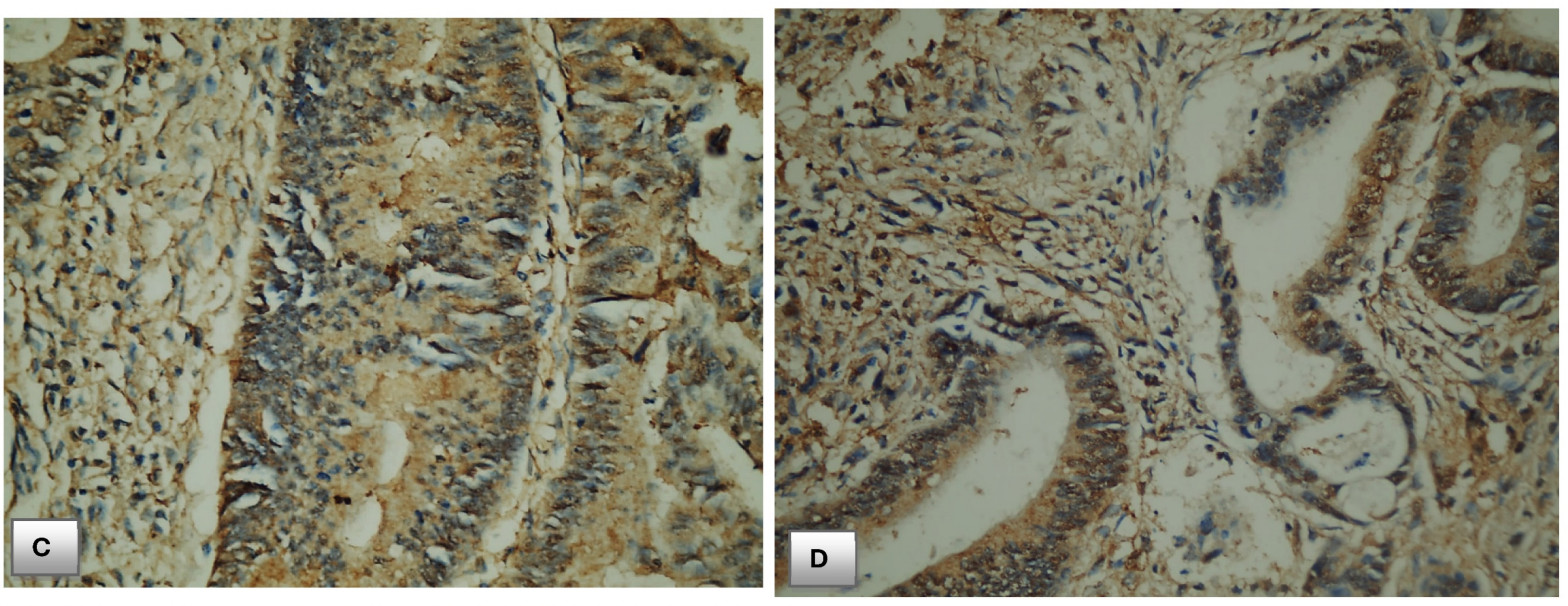

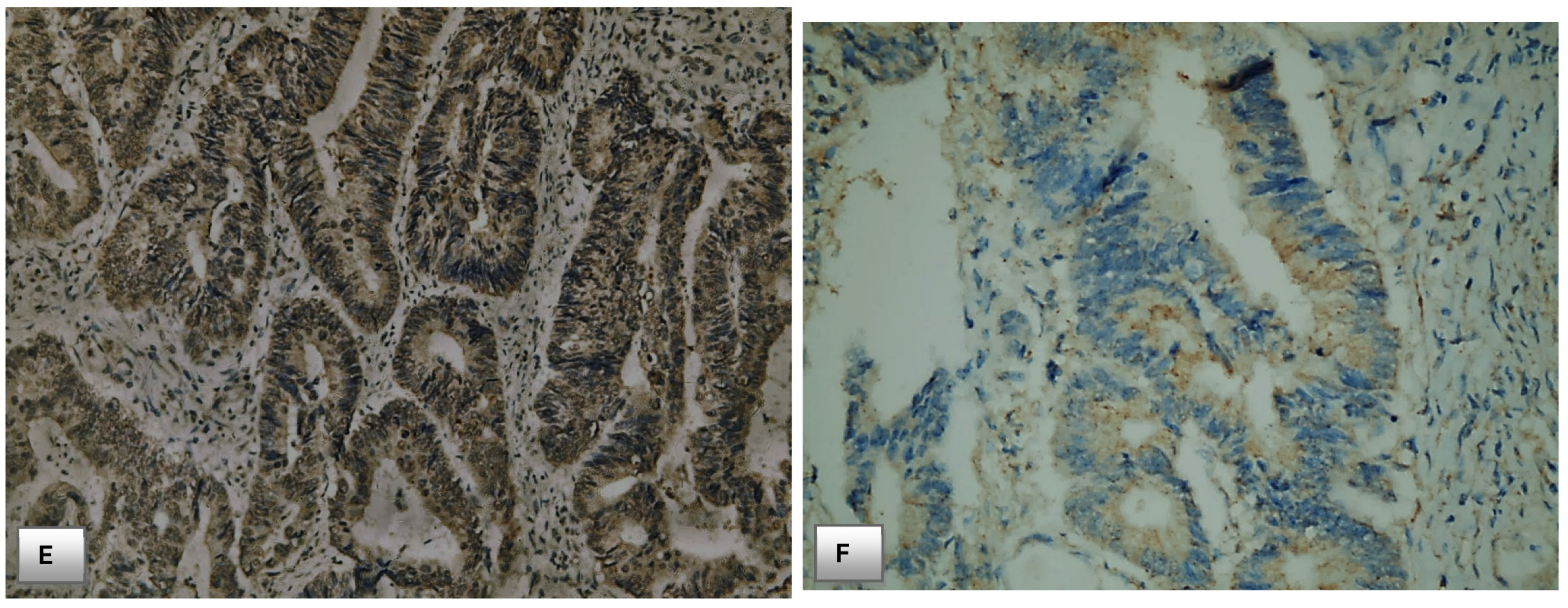

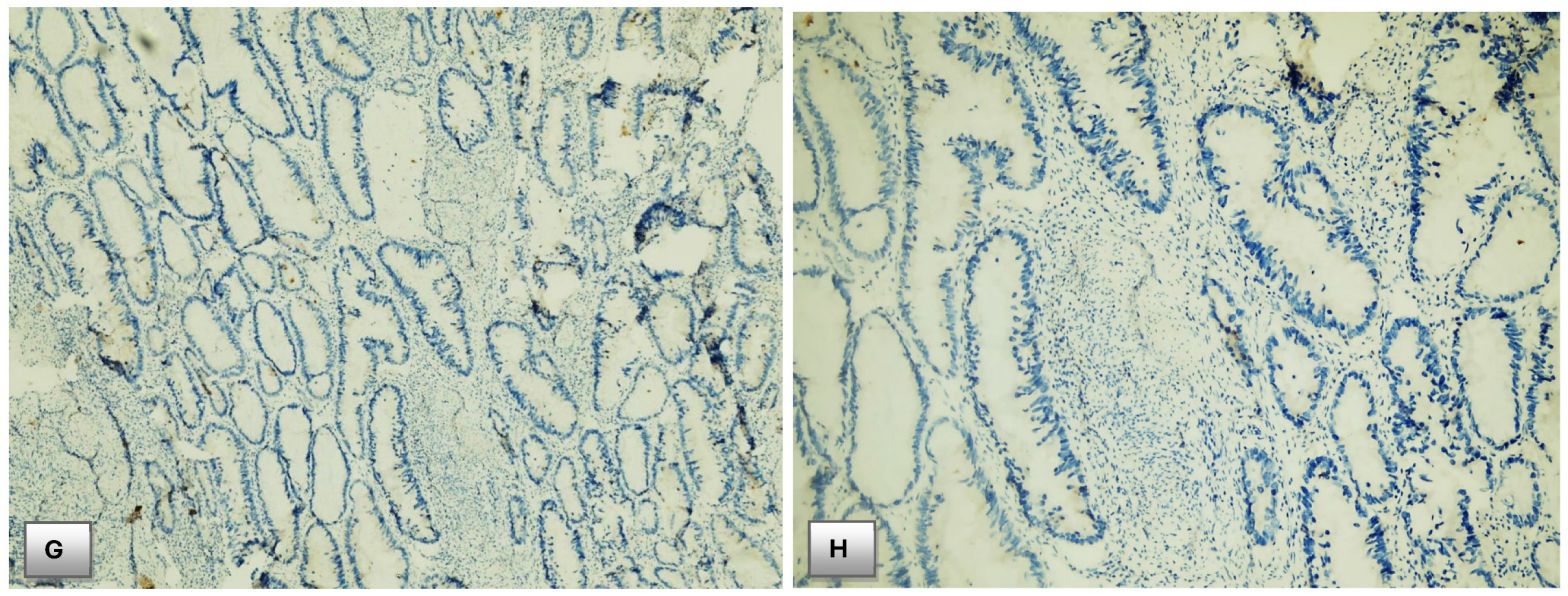
Immunohistochemical expression of PTEN. (A) PTEN expression in normal colonic mucosa (x200). (B) Cytoplasmic expression of PTEN in normal colonic mucosa (x400). (C) Diffuse cytoplasmic staining of PTEN in case of colorectal adenocarcinoma (x400). (D) Strong cytoplasmic PTEN expression in non-metastatic case of colorectal adenocarcinoma (x200). (E) A case of stage II adenocarcinoma showing strong PTEN expression (x200). (F) Adenocarcinoma stage III with mild PTEN expression (x400). (G) Metastatic case of colorectal adenocarcinoma with negative PTEN expression (x100). (H) Metastatic case of colorectal adenocarcinoma with negative PTEN expression (x200).

However, the expression was weekly positive in **4 (20%)** of cases of CRC stage **IV** with no cases detected as moderately positive nor strongly positive in this **stage IV** group. In the light of those findings, absent of PTEN staining positively associated with advanced stage of CRC and carry a bad outcome, while normal PTEN expression or slight decrease in PTEN activity positively associated with early stage of cancer with significant p. value **(p<0.001)**. **Table 5, Fig. 4.**

## Discussion

More than half million people die from colorectal cancer each year worldwide, being the most frequent malignant illness of the gastrointestinal tract, the second most prevalent cancer in women and the third most common in men 10.

The rectum alone is home to one-third of all colorectal malignancies. Once colorectal cancer has been histologically diagnosed, it is necessary to assess the disease's local and distant spread. Nearly one-fourth of those with colorectal cancer who recently got a diagnosis have distant metastases 11.

The main determinants of a person's risk for colorectal cancer are exposure to risk factors, which is potentially adjustable, as well as non-modifiable dispositional characteristics like age, sex, and family history. According to estimates, unhealthy habits such as smoking, consuming large amounts of red and manufactured meat, being overweight, having diabetes, and drinking too much alcohol account for half of the chance of developing colorectal cancer 12.

PTEN is one of the most common cancer suppressor genes in human malignancies. It plays a vital function in the PTEN/PI3K/AKT pathway, and the loss of PTEN could lead to higher quantities of phosphatidylinositol (3,4,5)-trisphosphate (PIP3). AKT and 3 phosphoinositide-dependent kinase 1 (PDK1) are both highly activated by the latter. AKT is a serine/threonine-specific protein kinase that is involved in several cellular processes, such as the metabolism of glucose, transcription of cells, proliferation, apoptosis, and migration 13.

PTEN expression or function can be compromised at several stages of colorectal carcinogenesis, including genomic, transcriptional, post-transcriptional, and post-translational 14. PTEN expression loss is thought to occur in more than one-third of CRC cases 15 and can be brought on by both genetic and epi-genetic causes 16.

Since the role of PTEN in the development of CRC is still a matter of debate, we attempted in this study to assess the significance of PTEN gene mutation and PTEN immunohistochemistry expression as risk factors of colorectal cancer development. According to our knowledge, our study is the first to examine PTEN's contribution to the emergence for colorectal cancer in Egyptian patients.

In this study, the mean age of our patients was 48 years at diagnosis, indicating a relatively younger age of onset for patients with CRC in Egypt. Most patients were males, with a positive family history of CRC in 6% of patients, and the predominant site of the lesion was the rectosigmoid part.

Our findings concur with those of research conducted in India on 970 CRC patients. Rectal cancer was found in 58.7%. There was an abundance of men. The average age was 47 years. There was a history of CRC in the family for 6.7% of patients with rectal cancer 17.

In accordance with PTEN genotypes and allele frequencies, our result showed no discernible variance in the frequencies of the TT, TC, and CC genotypes in CRC patients compared to controls. Additionally, no noticeable difference has been detected between the T allele and C allele frequencies between the two study groups. Even the mutant CC genotype and C allele were higher in cancer cases, but no significant difference has been detected compared with controls.

These findings suggest that there may be no association between genotypes TC+CC and C alleles and the risk of CRC development among Egyptian patients.

On the other hand, a highly significant difference was detected when comparing immunohistochemical PTEN activity between CRC cases and controls. The expression of PTEN in normal colorectal mucosa was higher than that in carcinoma cases, with low PTEN activity and loss of its expression in 60% of patients with CRC. Those results were in line with the results of *Sun and his colleagues,* who found that increased expression of PTEN was found in normal colorectal mucosa and adenoma cases compared to carcinoma ones 9. Also, *Yazdani et al.* and *Serebriiskii et al.* found a highly statistically significant difference in immunohistochemical PTEN expression between cancerous and non-cancerous colorectal tissues 18-19.

This finding suggests a significant association between loss of PTEN activity and the risk of CRC development.

PTEN is a phosphatase-active tumor suppressor gene. Phosphatidylinositol-trisphosphate is dephosphorylated by the PTEN protein, which functions as a phosphatase. PTEN selectively catalyzes the dephosphorylation of the inositol ring's 3′ phosphate in PIP3, generating the biphosphate product PIP2, and preventing the phosphorylation of phosphatidyl-inositol 3-kinase, which hinders the activity of Akt (protein kinase B) and its associated kinases and triggers apoptosis 20.

Our results revealed no association between PTEN expression and age, sex, the location of the lesion, the histological type, or the histological grade of cancer.

This is supported by a study by *Mirzapour et al.* that examined 151 tissue samples from CRC patients utilizing immunohistochemistry PTEN labeling 21. Additionally, *Colakoglu et al.'s* investigation of 76 patients with primary CRC observed no correlation between immunohistochemistry PTEN expression and age, sex, or site of cancer 22. On the other hand, *Molinari et al.* observed that PTEN expression was lower in distal colorectal malignancies than proximal carcinomas and suggested that this finding may be related to distinct biological processes that underneath the development of proximal and distal sporadic CRCs. Malignancies in the proximal colon often exhibit microsatellite instability, whereas malignancies of the distal colon and the rectum frequently exhibit chromosomal instability 16. Also, the study of *Atreya et al.* found a significant correlation between PTEN immunohistochemical expression and grade of colorectal carcinoma, this disparity could be attributed to a lack of standardization in terms of both staining technical performance and analytic approaches 23.

There was statistically significant correlation between immunohistochemical PTEN expression and depth of tumor invasion (T stage) of CRC cases, PTEN expression de-creased with increased T stage and loss of its expression was detected in T3 cases. This was in line with the results of the study performed by *Waniczek* who found decreased and absence of PTEN expression in T3 and T4 cases respectively 24. Also, *Serebriiskii* and his team found the same association between PTEN expression profiles and depth of tumor invasion 19.

This work has shown substantial associations between lymph node metastases and advanced TNM stage and absence of PTEN expression as well as low positive PTEN activity.

This agrees with the study results of *Mirzapour et al*. who found a significant association between nodal metastases and advanced TNM stage and negative PTEN expression in patients with CRC 20. In addition, *Waniczek et al.* also correlated decreased PTEN expression with advanced TNM stage, however he found no association between PTEN ex-pression and nodal status, and this difference may be due to the difference in sample size 24. Furthermore, *Atreya et al.* reported that loss of PTEN expression was linked to CRC metastases and a poor prognosis in their research of 63 patients with CRC 23.

*Yazdani et al.* also found that absent and decreased PTEN expression occurs with advanced stage of CRC, his study revealed that 66 out of 100 CRC cases staged as III and IV stages showed negative and weekly positive PTEN expression 18.

A potential link of PTEN expression on CRC development and progression was noticed in this study which showed nearly 80% of patients with lost PTEN activity was stage IV CRC, while, no one of patients with strong PTEN activity was stage IV, and nearly 26% of patients with strong PTEN activity was stage I CRC.; with higher PTEN expression levels found in patients with early-stages CRC (AJCC stages I&II) and lower expression levels or even negative PTEN staining found in patients with advanced stages of cancer (AJCC stag III& IV). On the contrary, there was no discernible relationship between the distribution of PTEN gene mutation and the AJCC staging and histological grading of colorectal cancer.

The findings of this investigation, together with a related study on 125 individuals with colorectal cancer, showed that PTEN loss is a significant contributor to the emergence and spread of colorectal cancer 18. Further research on 39 patents revealed that the development of CRC was ultimately caused by a progressive reduction in PTEN expression. Additionally, decreased PTEN activity in CRC increases the likelihood of advancement and triggers apoptosis. The proliferation of cancerous cells is prevented by PTEN upregulation 25.

According to *Sawai et al.'s* results, PTEN may be important for prognosis in colorectal cancer patients. Those with low PTEN expression or PTEN loss may see an increase in invasion and even metastasis 26.

On the other hand,* Mao and colleagues* discovered no correlation between PTEN loss and metastatic colorectal cancer. This could be connected to racial variations that influence genetic mutation 27.

Like our study, The PTEN gene mutation was not linked to an increased risk of developing CRC in research that included 421 patients with CRC and 483 healthy controls 28.

*Ghiţă C et al.* discovered that tumor suppressor gene mutations may be present in colorectal cancer based on their investigation of 22 people diagnosed with colorectal cancer. The degree to which these mutations are expressed varies, which may account for the patients' varying prognoses 29.

In addition, *Molinari et al.,* considered that the use of PTEN as a prognostic or predictive marker in CRC is also currently under discussion, for several reasons, including: (1) the scope of the study; (2) the criteria for inclusion for patients; and (3) the techniques used to measure PTEN alteration 16.

### Study limitations

Relatively small sample size and lack of patient follow-up.

## Conclusions

PTEN gene mutation may has no role in colorectal cancer development and progression among Egyptian patients. However, loss of immunohistochemical PTEN expression or weak activity may play a role in CRC development and its progression. To corroborate such a result, more research is required.

## Figures and Tables

**Table 1:** Demographic data and laboratory data of studied groups

Variable	Cases (N= 100)	Control (N= 50)	P- value
Age **(mean± SD)**	48.87±12.91	47.88±11.76	0.649
Sex **(N%)**			0.064
**Male**	60 (60%)	22 (44%)	
** Female**	40 (40%)	28 (56%)	
Smoking **(N%)**	20 (20%)	6 (12%)	0.222
Presenting manifestation **(N%)**			
**Bleeding per-rectum**	25 (25%)		-------
**Abdominal pain**	18 (18%)		
**Chronic constipation**	25 (25%)		
**Intestinal obstruction**	8 (8%)	-------	
**Weight loss**	22 (22%)		
**Anemia**	17 (17%)		
**Chronic diarrhea**	0 (0%)		
Positive family history of colorectal cancer **(N%)**	6 (6 %)	-----	------
Positive family history of cancer other than colorectal cancer **(N%)**	4 (4%)	-------	-------
Site of lesion **(N%)**			
**Caecum**	20 (20%)		
**Ascending colon**	17 (17%)		
**Descending colon**	15 (15%)	-------	--------
**Rectosigmoid**	48 (48%)		
PTEN (rs701848) genotype **(N%)**			
**TT**	10 (10%)	6 (12%)	
**TC**	60 (60%)	30 (60%)	0.918
**CC**	30 (30%)	14 (28%)	
CBC			
**Hemoglobin (mean± SD)**	10.4±1.62	11.14±1.49	**0.008**
**WBCs**			
**(mean± SD)**	8.44±7.47	6.65±2.79	**0.011**
**Median (IQ range)**	6.76(5.9-9.3)	5.8(4.85-7.73)	
**Platelets (mean± SD)**	272.18±90.12	318.52±99.08	**0.00**
Creatinine **(mean± SD)**	0.89±0.21	0.88±0.14	0.845
Prothrombin time			
**(mean± SD)**	12.46±1.21	12.17±0.81	0.352
**Median (IQ range)**	12.2 (11.5-13.18)	11.65 (11.5-13)	
Prothrombin concentration			
**(mean± SD)**	93.61±10.82	94.92±7.26	0.748
**Median (IQ range)**	99 (88.6-100)	99.25 (92.6-100)	
INR			
**(mean± SD)**	1.06±0.1	1.06±0.08	0.436
**Median (IQ range)**	1(1-1.09)	1.02 (1-1.1)	
Liver function tests			
ALT			
**(mean± SD)**	26.2±15.42	20.42±6.49	0.071
**Median (IQ range)**	21.5(17-31)	19.5(16-22.5)	
AST			
**(mean± SD)**	22.89±12.12	23.72±8.93	0.114
**Median (IQ range)**	19(16-25.75)	21(17-29)	
Albumin			
**(mean± SD)**	3.83±0.64	4.22±0.57	**<0.001**
**Median (IQ range)**	3.8 (3.5-4.1)	4.1(3.9-4.7)	
Bilirubin			**<0.001**
**(mean± SD)**	1.56±2.03	0.91±0.34	
**Median (IQ range)**	1.1 (0.96-1.7)	0.99 (0.67-1.1)	

**Table 2:** Genotypes and allele frequencies of PTEN (rs701848) genotype among study groups

Study groups	Variables																	
	PTEN (rs701848) genotype														PTEN (rs701848) alleles			
	TT		TC		CC		TT+TC		CC		TT		TC+CC		T		C	
	No.	%	No.	%	No.	%	No.	%	No.	%	No.	%	No.	%	No.	%	No.	%
Cases **(N=100)**	10	10	60	60	30	30	70	70	30	30	10	10	90	90	80	40	120	60
Controls **(N=50)**	6	12	30	60	14	28	36	72	14	28	6	12	44	88	42	42	58	58
P-value **(χ^2^)**	0.918(0.170)						0.800(0.064)				0.708(0.140)				0.740(0.111)			
OR **(95%CI)**	------						0.907(0.428-1.923)				0.815(0.278-2.386)				0.921(0.565-1.499)			

**Table 3:** PTEN (rs701848) genotypes and allele frequencies with the histologic grading and AJCC staging of CRC

PTEN (rs701848) genotype and alleles	Histologic grade I & II (n=77)	Histologic grade III (n=23)	P value
TT	5(6.5%)	5(21.7%)	0.102
TC	48(62.3%)	12(52.2%)	
CC	24(31.2%)	6(26.1%)	
T- allele	58(37.7%)	22(47.8%)	0.288
C- allele	96(62.3%)	24(52.2%)	
**PTEN (rs701848) genotype and alleles**	**AJCC (I&II) (n= 42)**	**AJCC (III & IV) (n= 58)**	**P value**
TT	1(2.4%)	9(15.5%)	0.092
TC	28(66.7%)	32(5 5.2%)	
CC	13(31%)	17(29.3%)	
T- allele	30(35.7%)	50(43.1%)	0.365
C- allele	54(64.3%)	66(56.9%)	

**Table 4:** PTEN expression in cases of Colorectal cancer and control

Cases	Number of cases	PTEN Expression				P- value
		Negative	Positive			
			1+	2+	3+	
Colorectal cancer	100	28 (28%)	32(32%)	20(20%)	20 (20%)	**<0.001**
Control	50	0	4 (8%)	16(32%)	30 (60%)	

P value was calculated by Chi-square test

**Table 5:** Association between PTEN expression and the clinicopathological parameters in CRC patients

Clinicopathological parameters	Number of CRC cases (100)	PTEN Expression				P-value
		Negative	Positive			
			1+	2+	3+	
		28 (28%)	32 (32%)	20 (20%)	20 (20%)	
**Age**	48.87±12.91	51.5±13.64	49.5±13.46	42.55±11.1	50.5±11.35	0.094
**Sex**						0.981
Male	60 (60%)	16 (35.7%)	20 (33.3%)	12 (20%)	12 (20%)	
Female	40 (40%)	12 (30%)	12 (30%)	8 (20%)	8 (20%)	
**Site of CRC**						0.438
Caecum	20 (20%)	2 (10%)	8 (40%)	3 (15%)	7 (35%)	
Ascending colon	17 (17%)	7 (41.17%)	6 (35.29%)	2 (11.76%)	2 (11.76%)	
Descending colon	15 (15%)	5 (33.33%)	5 (33.33%)	3 (20%)	2 (13.33%)	
Rectosigmoid	48 (48%)	14 (29.16%)	13 (27.08%)	12 (25%)	9 (18.75%)	
**Histological Grade**						0.375
I	33 (33%)	8 (24.2%)	13 (39.4%)	6 (18.2%)	6 (18.2%)	
II	44 (44%)	10 (22.7%)	12 (27.3%)	12(27.3%)	10(22.7%)	
III	23 (23%)	10 (43.5%)	7 (30.4%)	2 (8.7%)	4 (17.4%)	
**Histological Type**						0.345
Conventional adenocarcinoma	70 (70%)	18 (25.7%)	26(37.1%)	14(20%)	12(17.1%)	
Mucinous carcinoma	30 (30%)	10 (33.3%)	6(20%)	6 (20%)	8 (26.7%)	
**T stage**						**0.027**
I	24 (24%)	4 (16.7%)	8 (33.3%)	4 (16.7%)	8 (33.3%)	
II	44 (44%)	8 (18.2%)	16(36.3%)	12(27.3%)	8 (18.2%)	
III	32 (32%)	16 (50%)	8(25%)	4 (12.5%)	4 (12.5%)	
**Lymph node metastasis**						**0.030**
N0	45 (45%)	6 (13.3%)	15 (33.3%)	14(31.1%)	10(22.2%)	
N1	30(30%)	14(46.7%)	8 (26.6%)	4 (13.3%)	4 (13.3%)	
N2	25(25%)	8 (32%)	9 (36%)	2 (8%)	6 (24%)	
**AJCC stage**						**<0.001**
** I**	34 (34%)	0 (0%)	15 (44.1%)	10(29.4%)	9 (26.5%)	
** II**	8 (8%)	3(37.5%)	0 (0%)	4 (50%)	1 (12.5%)	
**III**	38 (38%)	9 (23.7%)	13(34.2%)	6 (15.8%)	10(26.3%)	
** IV**	20 (20%)	16 (80%)	4 (20%)	0 (0%)	0 (0%)	

P value was calculated by Chi-square test

## Abbreviations

PTEN: phosphatase and tensin homolog
CRC: Colorectal cancer
AJCC: American Joint Committee on Cancer staging system
PIP3: phosphatidylinositol (3,4,5)-trisphosphate
PDK1: 3 phosphoinositide-dependent kinase 1
AKT: serine/threonine kinase
